# Reactive oxygen species and transcript analysis upon excess light treatment in wild-type *Arabidopsis thaliana *vs a photosensitive mutant lacking zeaxanthin and lutein

**DOI:** 10.1186/1471-2229-11-62

**Published:** 2011-04-11

**Authors:** Alessandro Alboresi, Luca Dall'Osto, Alessio Aprile, Petronia Carillo, Enrica Roncaglia, Luigi Cattivelli, Roberto Bassi

**Affiliations:** 1 Dipartimento di Biotecnologie, Università di Verona, Strada Le Grazie 15, I - 37134 Verona, Italy; 2 CRA Centro di Ricerca per la Genomica, Via San Protaso 302, 29017 Fiorenzuola d'Arda, Italy; 3 Dipartimento di Scienze della Vita, Seconda Università degli Studi di Napoli, Via Vivaldi 43, Caserta, Italy; 4 Dipartimento di Scienze Biomediche, Università di Modena e Reggio Emilia, Via Campi 287, 41100 Modena, Italy

## Abstract

**Background:**

Reactive oxygen species (ROS) are unavoidable by-products of oxygenic photosynthesis, causing progressive oxidative damage and ultimately cell death. Despite their destructive activity they are also signalling molecules, priming the acclimatory response to stress stimuli.

**Results:**

To investigate this role further, we exposed wild type *Arabidopsis thaliana *plants and the double mutant *npq1lut2 *to excess light. The mutant does not produce the xanthophylls lutein and zeaxanthin, whose key roles include ROS scavenging and prevention of ROS synthesis. Biochemical analysis revealed that singlet oxygen (^1^O_2_) accumulated to higher levels in the mutant while other ROS were unaffected, allowing to define the transcriptomic signature of the acclimatory response mediated by ^1^O_2 _which is enhanced by the lack of these xanthophylls species. The group of genes differentially regulated in *npq1lut2 *is enriched in sequences encoding chloroplast proteins involved in cell protection against the damaging effect of ROS. Among the early fine-tuned components, are proteins involved in tetrapyrrole biosynthesis, chlorophyll catabolism, protein import, folding and turnover, synthesis and membrane insertion of photosynthetic subunits. Up to now, the *flu *mutant was the only biological system adopted to define the regulation of gene expression by ^1^O_2_. In this work, we propose the use of mutants accumulating ^1^O_2 _by mechanisms different from those activated in *flu *to better identify ROS signalling.

**Conclusions:**

We propose that the lack of zeaxanthin and lutein leads to ^1^O_2 _accumulation and this represents a signalling pathway in the early stages of stress acclimation, beside the response to ADP/ATP ratio and to the redox state of both plastoquinone pool. Chloroplasts respond to ^1^O_2 _accumulation by undergoing a significant change in composition and function towards a fast acclimatory response. The physiological implications of this signalling specificity are discussed.

## Background

Plant growth is inhibited by many forms of abiotic stress, including intense light [1], nitrogen and phosphorus starvation [2,3], water stress/high salinity [4] and extreme temperatures [5,6]. Excess light induces the re-organization of the photosynthetic apparatus to facilitate light harvesting while avoiding potentially damaging effects. Concomitantly, metabolism is redirected towards the synthesis of protective compounds such as flavonoids [7,8], tocopherol and carotenoids [9,10], which participate directly in stress responses.

The chloroplast is a crucial intersection for environmental stimuli [11-13]. Short-term responses to excess light, elicited in a timeframe of seconds to minutes, include enhanced thermal dissipation of light energy [14-16] and detachment of the outer antenna system from the photosystem II (PSII) reaction centre [17,18]. Longer-term acclimation responses include an increase in the PSI/PSII ratio, and the production of Rubisco, cytochrome b_6_/f complexes and ATPase at higher levels in order to increase the rate of O_2 _evolution under saturating light conditions and avoid plastoquinone (PQ) over-reduction. Moreover, the capacity for thermal energy dissipation (Non-Photochemical Quenching, NPQ) increases as PsbS accumulates [19,20].

Although cytochrome b_6_/f, ATPase and Rubisco are encoded by chloroplast genes, the vast majority of plastid polypeptides are encoded by nuclear genes and are imported as precursors through the plastid envelope [21,22]. Acclimatory responses therefore require the coordinated regulation of plastid and nuclear genes, which involves a retrograde signal [12,23-27]. In the last decade transcriptome analysis has confirmed the importance and sophistication of this regulatory network [13,28-30], but the signals and transduction pathways are not yet fully understood. Proposed signalling molecules include Mg-protoporphyrin IX [31], which couples the rate of chlorophyll synthesis to the expression of nuclear-encoded pigment-binding proteins, and the redox equilibrium of plastoquinone (PQ/PQH_2_) [32]. However, Mg-protoporphyrin IX is absent under conditions leading to the repression of nuclear genes [33], and only 54 nuclear genes appear to be controlled by the PQ redox state and photosynthetic electron flow (PEF) [34], casting doubt on their proposed role. Furthermore, analysis of the barley *viridis zb63 *mutant (which has a constitutively reduced PQ pool) suggests that the expression of photosynthesis-related genes is not coupled to the redox state of PQ [35].

Reactive oxygen species (ROS) have recently been proposed as candidate signalling molecules in acclimation because they can modulate gene expression when added to cell culture media, and gene expression patterns are altered in mutants accumulating higher or lower levels of ROS [36-38]. Although renowned for the damage they cause to proteins, lipids and nucleic acids [39], ROS also have several important physiological functions such as defence against pathogens [40] and the regulation of plant development [41-43]. Plants have evolved a complex regulatory network to mediate abiotic stress responses based on ROS synthesis, scavenging and signalling, although more work is needed to decipher the signalling pathways and the crosstalk between them [36,44,45]. Signals representing environmental changes are the first important step leading to plant acclimation and survival [37].

We exposed *Arabidopsis thaliana *plants to intense light at low temperatures, which strongly inhibits photosynthetic electron flow and reduces PSII efficiency, leading to the over-excitation of pigments and the accumulation of singlet oxygen (^1^O_2_), a peculiar ROS species that is the first excited electronic state of molecular oxygen [46]. We compared wild-type plants to the double mutant *npq1lut2*, which lacks violaxanthin de-epoxidase (VDE) and lycopene-ε-cyclase (LUT2) activities, and therefore cannot synthesize two major photoprotective xanthophylls: lutein and zeaxanthin. These molecules help to quench chlorophyll triplet states (^3^Chl*) and scavenge ^1^O_2 _released within the thylakoid membrane [47,48]. Due to the defect in xanthophyll composition, the *npq1lut2 *mutant exhibits a remarkable sensitivity to high light [49] and accumulates higher levels of ^1^O_2 _than wild-type plants, while the accumulation of other ROS is unaffected as are other putative retrograde signals such as the PQ redox state and the ATP/ADP ratio. The system that gave a great breakthrough in the study of ^1^O_2 _accumulation in plants is the conditional *flu *mutant. This mutant in the dark accumulates protochlorophyllide that acts as a photosensitizer upon illumination and generates ^1^O_2 _in the stroma of chloroplasts [50]. In *flu*, ^1^O_2 _accumulation mediates the activation of a stress response [29] that is different from those induced by other ROS such as superoxide anion (O_2_^-^) or hydrogen peroxide (H_2_O_2_) [30]. Further results showed that Executer1/2 are chloroplast proteins crucial for ^1^O_2_-mediated stress responses [51]. However, xanthophyll mutants have been recently used to study the effect and the signalling pathway of ^1^O_2_[46,52]. We are clearly dealing with two different systems that accumulate ^1^O_2_. The most studied that depends on ^1^O_2 _steady-state accumulation from chlorophyll precursors and the second one that depends on the photoprotective activity of xanthophylls in thylakoid membranes. In the first case the toxic effect of ^1^O_2 _has a major role in defining the phenotype, while in *npq1lut2 *its effect as signal molecule is more important. We applied stress conditions within a physiological range, leading to acclimation rather than the apoptotic responses reported in previous studies [30,53]. By limiting cross-talk between the apoptotic and acclimatory signal transduction pathways, we found that ^1^O_2 _can function as a signal in both wild-type and *npq1lut2 *mutants under oxidative stress.

## Results

### Genes regulated by intense light at low temperatures in wild-type and mutant plants

**Figure 1:**
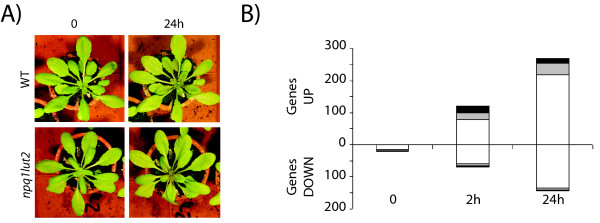
**Effect of high light treatment on plant growth and on gene expression at low temperature**. A) Phenotype of WT and *npq1lut2 *plants at time 0 and 24 h under the high-light and low temperature conditions specified in the text. B) Number of genes (probe set) up or down regulated in *npq1lut2 *mutant compared to control wild-type plants at the three time points of the experiment (0, 2 and 24 hours of treatment at 10°C and 1000 μmol photons m^-2 ^s^-1^). White panels are genes from at least 1-fold change to 2-fold change difference, grey panels are genes from at least 2-fold change to 3-fold change difference and black panels are genes with at least a 3-fold change between the mutant and the control. Fold change is indicated in a log_2 _scale.

An Affymetrix GeneChip^® ^Arabidopsis ATH1 Genome Array was used to compare the transcriptional footprints of wild-type *Arabidopsis thaliana *plants and the *npq1lut2 *mutant when both were transferred at 10°C and exposed to either very low light levels (time 0, before the application of stress) or very high light levels (1000 μmol m^-2 ^s^-1^) for 2 or 24 h (Figure 1). Three biological replicates were analyzed in each treatment group. These conditions (low temperature associated to high light) were carefully chosen in order to emphasize the effect caused by the lack of the two photoprotective xanthophylls [47].

We noted that many genes were similarly regulated by light at low temperatures regardless of the genetic background, i.e. they were not influenced by the mutations. We have first compared different time points for each genotype to identify genes responding in the same way in both genotypes. These genes represent the response to high-light and low-temperature conditions in our experiment. Among the rapidly-responding genes (reaction to stress within 2 h), 812 were modulated in both wild-type and mutant plants, all showing the same directional response in both backgrounds (476 up-regulated and 336 down-regulated; Additional file 1: Table S1). Among the delayed-response genes (reaction to stress within 24 h), 1128 genes were modulated in both backgrounds, again all showing the same directional response (611 up-regulated and 517 down-regulated; Additional file 1: Table S2).

Functional classification of the above genes was carried out using FunCat version 2.1 [54] and the most significant results (p < 0.005) are summarized in Table 1. A complete list with subcategories is provided in Additional file 1: Table S3. Many of the genes (up-regulated and down-regulated) fell into the Cell Rescue, Interaction with Cell Environment and Interaction with the Environment categories, which are generally associated with stress responses or hormone signalling. Among the down-regulated genes, there was a significant over-representation of those in the Control of Transcription and Cell Wall Biogenesis functional categories, whereas many genes involved in Primary and Secondary Metabolism were up-regulated (176 after 2 h, 210 after 24 h). For example, a change in L-phenylalanine metabolism, reflecting the overexpression of chloroplast chorismate mutase (AT3G29200; *CM1*) and phenylalanine ammonia-lyase 1 (AT2G37040; *PAL1*), could serve as a secondary pathway for the synthesis of phenylpropanoids and flavonoids. Additional file 1: Table S3 shows that photosynthesis, energy conversion and regeneration, and light absorption are down-regulated after 24 h, possibly because energy pathways are overloaded and therefore feedback-inhibited when constantly exposed to intense light.

**Table 1:** Functional classification of genes regulated by intense light at 10°C.

Functional Categories	UP 2 h (476)	UP 24 h (606)	DOWN 2 h (336)	DOWN 24 h (370)
01 Metabolism	19.6 (176)	17.3 (210)	-	-
02 Energy	-	2.2 (23)	-	-
11 Transcription	-	-	7.5 (57)	4.5 (72)
14 Protein Fate	5.4 (78)	-	-	-
16 Binding Function	7.0 (151)	5.3 (182)	-	-
20 Transport	3.8 (59)	-	-	-
30 Signal Transduction	-	-	-	2.9 (38)
32 Cell Rescue	14.1 (91)	7.99 (79)	-	4.1 (47)
34 Interaction with Cell Environment	12.4 (87)	6.89 (77)	7.2 (44)	9.5 (79)
36 Interaction with the Environment	7.2 (47)	-	3.9 (22)	6.3 (43)
40 Cell Fate	-	-	-	3.7 (27)
70 Subcellular Localization	-	16.7 (326)	10.6 (160)	-

Function and cellular localization of genes regulated by intense light (1000 μmol photon m^-2 ^s^-1^, 10°C) in both wild-type and *npq1lut2 *mutant plants. Functional categories and their consistency were defined using MIPS functional catalogue (p ≤ 0.005). Up-regulated and down-regulated genes were analyzed after 2 and 24 h stress. For each subset, the number of genes is shown in brackets.

The ten most strongly modulated genes after 2 h included several with a regulatory function, which are likely to be involved in the activation of a stress response according to their GENEVESTIGATOR response profiles (Additional file 1: Table S1). These comprised three transcription factors (AT4G28140, AT1G56650 and AT2G20880), two heat shock proteins (AT3G12580 and AT2G20560) and one putative allene oxide cyclase (AT3G25780). After 24 h we observed the strong induction of genes known or suspected to be involved in flavonoid biosynthesis or modification, i.e. dihydroflavonol 4-reductase, *DFR*, AT5G42800; anthocyanin 5-aromatic acyltransferase, *AAT1*, AT1G03940-AT1G03495; anthocyanin pigment 2 protein, *PAP2*, AT1G66390; anthocyanin 5-O-glucosyltransferase, AT4G14090; flavonoid 3'-hydroxylase, *F3'H*, AT5G07990; MYB family transcription factor, *MYB75/PAP1*, AT1G56650; UDP-glucosyl transferase, AT5G54060; and anthocyanidin synthase, AT4G22870 (Additional file 1: Table S2). These genes are known to be important checkpoints in flavonoid biosynthesis as shown by microarray experiments performed under various abiotic stress conditions [7].

### Genes regulated by intense light at low temperatures in mutant plants only

Only 20 genes were found to be differentially expressed when unstressed wild type and mutant plants were compared (18, considering that two of them are responsible for *npq1lut2 *mutation). All 18 genes were down-regulated in the mutant, suggesting that the two backgrounds are metabolically very similar when there is no stress and that the 18 genes may be directly influenced by the lack of NPQ1 and LUT2 enzyme activities, or of the corresponding products (Figure 1).

Following exposure to intense light, the number of differentially expressed genes increased dramatically. After 2 h, 121 genes were up-regulated in the mutant and 69 down-regulated, and after 24 h, 270 genes were up-regulated and 144 down-regulated (Figure 1). The distribution of functional categories among these genes was similar to the genes modulated in the same manner in both backgrounds. However, a distinct group of 67 genes specifically repressed in the wild type plants after 2 h of stress but not repressed in the mutant (p = 1.12 × 10^-9^) was shown to encode chloroplast proteins (Table 2), 38 with no known function and others identified as transcription factors and pentatricopeptide repeat-containing proteins (PPR), possibly participating in ROS signal transduction from the chloroplast to the nucleus and vice versa [55]. This indicates that nuclear gene expression might be influenced by carotenoid composition and anti-oxidant activity in thylakoid membranes, especially when plants are placed under oxidative stress.

**Table 2:** Expression of genes down-regulated in response to intense light at low temperature exclusively in wild-type plants (2 h time point).

Probeset	Locus identifier	Description	WT 0vs2
265067_at	AT1G03850	glutaredoxin family protein	-2,83
264379_at	AT2G25200	expressed protein	-2,67
248606_at	AT5G49450	bZIP family transcription factor	-2,04

249932_at	AT5G22390	expressed protein	-1,96
253305_at	AT4G33666	expressed protein	-1,74
263674_at	AT2G04790	expressed protein	-1,62
261196_at	AT1G12860	basic helix-loop-helix (bHLH) family	-1,51
256698_at	AT3G20680	expressed protein	-1,48
263209_at	AT1G10522	expressed protein	-1,48
248285_at	AT5G52960	expressed protein	-1,37
249750_at	AT5G24570	expressed protein	-1,35
247574_at	AT5G61230	ankyrin repeat family protein	-1,34
266899_at	AT2G34620	mitochondrial transcription factor-related	-1,34
261118_at	AT1G75460	ATP-dependent protease La (LON)	-1,31
263712_at	AT2G20585	expressed protein	-1,27
248795_at	AT5G47390	myb family transcription factor	-1,27
263593_at	AT2G01860	pentatricopeptide (PPR) repeat-containing	-1,26
254688_at	AT4G13830	DNAJ heat shock N-terminal (J20)	-1,24
261296_at	AT1G48460	expressed protein	-1,24
257615_at	AT3G26510	octicosapeptide/Phox/Bem1p (PB1)	-1,24
265457_at	AT2G46550	expressed protein	-1,23
249472_at	AT5G39210	expressed protein	-1,23
252136_at	AT3G50770	calmodulin-related protein	-1,21
252922_at	AT4G39040	expressed protein	-1,20
267591_at	AT2G39705	expressed protein	-1,20
257856_at	AT3G12930	expressed protein	-1,20
263264_at	AT2G38810	histone H2A	-1,19
249929_at	AT5G22340	expressed protein	-1,18
266329_at	AT2G01590	expressed protein	-1,18
248762_at	AT5G47455	expressed protein	-1,17
246506_at	AT5G16110	expressed protein	-1,17
258250_at	AT3G15850	similar to delta 9 acyl-lipid desaturase (ADS1)	-1,15
258683_at	AT3G08760	protein kinase family	-1,15
259013_at	AT3G07430	YGGT family protein	-1,14
253635_at	AT4G30620	expressed protein	-1,14
246033_at	AT5G08280	hydroxymethylbilane synthase	-1,14
248404_at	AT5G51460	trehalose-6-phosphate phosphatase (TPPA)	-1,13
248402_at	AT5G52100	dihydrodipicolinate reductase family protein	-1,13
256728_at	AT3G25660	glutamyl-tRNA(Gln) amidotransferase	-1,12
248663_at	AT5G48590	expressed protein	-1,12
245984_at	AT5G13090	expressed protein	-1,12
250663_at	AT5G07110	prenylated rab acceptor (PRA1)	-1,11
254011_at	AT4G26370	antitermination NusB domain	-1,10
261439_at	AT1G28395	expressed protein	-1,10
259889_at	AT1G76405	expressed protein	-1,10
253233_at	AT4G34290	SWIB complex BAF60b domain	-1,10
259976_at	AT1G76560	CP12 domain-containing	-1,10
260465_at	AT1G10910	pentatricopeptide (PPR) repeat	-1,10
246205_at	AT4G36970	remorin family protein	-1,09
257706_at	AT3G12685	expressed protein	-1,09
267219_at	AT2G02590	expressed protein	-1,08
264546_at	AT1G55805	BolA-like family protein	-1,08
258189_at	AT3G17860	expressed protein	-1,08
245877_at	AT1G26220	GCN5-related N-acetyltransferase (GNAT)	-1,06
266889_at	AT2G44640	expressed protein	-1,05
264963_at	AT1G60600	Phyllo- and plastoquinone biosynthesis	-1,05
260982_at	AT1G53520	chalcone-flavanone isomerase-related	-1,04
250529_at	AT5G08610	DEAD box RNA helicase (RH26)	-1,04
246294_at	AT3G56910	expressed protein	-1,04
249694_at	AT5G35790	Plastidic glucose-6-phosphate dehydrogenase	-1,03
266264_at	AT2G27775	expressed protein	-1,02
245494_at	AT4G16390	chloroplastic RNA-binding protein P67	-1,02
250353_at	AT5G11630	expressed protein	-1,02
248688_at	AT5G48220	Indole-3-glycerol phosphate synthase	-1,01

259738_at	AT1G64355	expressed protein	-1,00
250097_at	AT5G17280	expressed protein	-1,00
254755_at	AT4G13220	expressed protein	-0,98

The table shows the subset of genes encoding chloroplast proteins. The ratio between treated and control plants is expressed as a log_2 _scale. For each sample, the average of three repetitions is presented.

Focusing on differences in expression levels (Additional file 1: Table S4), we noticed that genes encoding heat-shock proteins (AT3G12580, AT5G51440 and AT1G59860-AT1G07400) were more strongly up-regulated in the mutant after 24 h, as were those encoding antioxidant proteins such as 2-alkenal reductase (*AER*; AT5G16970), which catalyzes the reduction of the α,β-unsatured bond of reactive carbonyls [56], methionine sulfoxide reductase 3 (*MSR3*; AT5G61640), which promotes thioredoxin-dependent reduction of oxidized methionine residues in ROS-damaged proteins [57], and the oxidative stress protein rubredoxin (AT5G51010) [58]. A squalene monooxygenase 1,1 gene (*SQP1,1*; AT5G24150) is 12x more strongly repressed in wild type plants than in mutants and might be the base for changes in plant morphology or oxidative stress response in HL conditions [59,60].

### Gene clustering

We next carried out a k-means cluster analysis, which organized all modulated genes into 11 clusters that differed little between wild-type and *npq1lut2 *(Additional file 2: Figure S1A). Therefore, an implemented cluster analysis was performed using a quality threshold algorithm (QT-clustering), in which we only considered genes with differences in transcript levels between the two genotypes at the three time points, i.e. 20 genes for time 0, 190 genes for time 2 h and 414 genes for time 24 h (Figure 1). The minimum number of probe-sets per cluster was fixed at 10, with a Pearson's correlation value fixed at 0.75. The number of clusters increased to 18, plus a group of 161 unclassified genes (Additional file 2: Figure 1B). Once again, there were few differences between the genotypes, with the exception of e.g. clusters 1, 3, 13 and 18. Cluster 18 attracted our attention because it showed the most striking difference between wild-type and *npq1lut2 *plants, and is strongly enriched in chloroplast genes (Table 3). Indeed, among the 80 probes in the *Arabidopsis *ATH1 Genome Array representing genes in the chloroplast genome (ATC codes), five belong to cluster 18. One of these genes encodes a protein hypothetically involved in PSI assembly (*AtYCF4*, ATCG00520), two encode photosystem core complex proteins, *PsbB *from PSII (D2; ATCG00270) and *PsaA *from PSI (ATCG00350), and two encode ATPase subunits (ATCG00130 and ATCG00140). Other genes in cluster 18 encode a transcription factor regulating the cryptochrome response (*AtCIB5*, AT1G26260), an L-ascorbate oxidase (AT4G39830), a kinase (AT1G21270) and two unknown proteins (AT1G23850 and AT2G46640). All these genes are modulated by intense light at low temperature in the wild-type, while there is no response in the mutant.

**Table 3:** Relevant cluster isolated by QT clustering.

	Locus identifier	FC	Description
245002_at	ATCG00270	-1,53	Encode PSII D2
245007_at	ATCG00350	-2,22	Encode PSI psaApsaB
245018_at	ATCG00520	-1,20	Hypothetical protein
245025_at	ATCG00130	-1,41	ATPase F subunit
245026_at	ATCG00140	-1,30	ATPase III subunit
245873_at	AT1G26260	-1,05	CIB5, bHLH
252862_at	AT4G39830	-2,50	L-ascorbate oxidase putative
259560_at	AT1G21270	-1,04	serine/threonine protein kinase 2 (WAK2)
263032_at	AT1G23850	-3,03	expressed protein
266320_at	AT2G46640	-1,01	expressed protein

This table shows the subset of genes in cluster 18. The ratio between *npq1lut2 *and wild-type plants after 2 h stress is expressed using a log_2 _scale. For each sample, the average of three repetitions is presented.

### ROS analysis in wild-type and npq1lut2 leaves

The *npq1lut2 *mutant was chosen because of its high sensitivity to photooxidative stress [47,49]. We determined the composition of ROS species released after the onset of illumination by infiltrating wild-type and mutant leaves with highly specific ROS-sensor probes: singlet-oxygen sensor green (SOSG) for ^1^O_2_, dichlorofluorescein (DCF) for H_2_O_2 _and OH., and proxyl-fluorescammine (ProxF) for O_2_^- ^and OH.[61]. All these probes show an increase in fluorescence emission in the presence of their specific trigger ROS, and the signal can be detected directly on the surface of an illuminated leaf using a fiber-optic fluorimeter. In particular, among all available probes specific for ^1^O_2_, we chose SOSG because, unlike other available fluorescent and chemiluminescent ^1^O_2 _detection reagents, it does not show any appreciable response to hydroxyl radical, H_2_O_2 _or superoxide anion; moreover, it was successfully applied to ^1^O_2 _detection in several systems, e.g. bacteria [62], diatoms [63], higher plants [48,63,64] and pigment-protein complexes isolated from higher plants [17,65]. Furthermore, C. Flors and co-workers applied SOSG to a range of biological systems that are known to generate ^1^O_2 _and in all cases, SOSG was confirmed as a useful *in vivo *probe for the detection of ^1^O_2_. Moreover, since highly sensitive probes for detection of H_2_O_2_, O_2_^- ^and OH.were also used in all measurements, any cross-detection of other ROS species than ^1^O_2 _by SOSG can be excluded.

**Figure 2:**
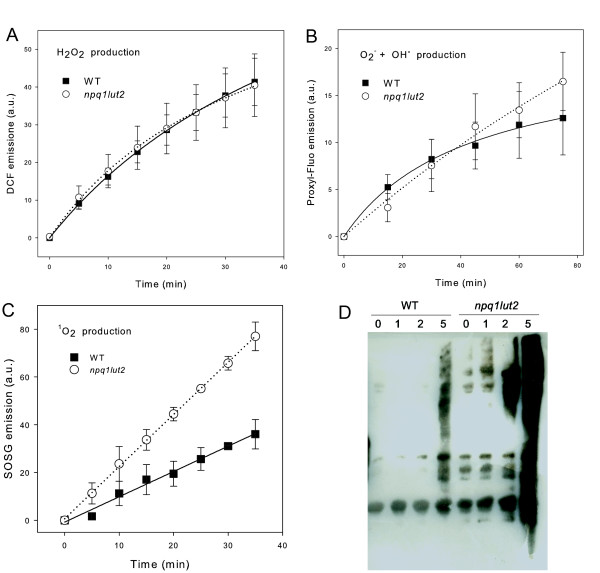
**Steady-state accumulation of ROS species and protein oxidizing activity in wild type and *npq1lut2 *mutant plants**. Specific probes were used to quantify the accumulation of several ROS in wild type and *npq1lut2 *detached leaves under stress (1000 μmol photons m^-2 ^s^-1^, 10°C). (A) DFC and (B) ProxF fluorescence was used to follow the accumulation of reduced forms of ROS. (C) SOSG fluorescence was used to follow singlet oxygen (^1^O_2_). Details on ROS measurements are given in material and method session. Symbols and error bars show means ± SD. (D) Western-blots were used to detect oxidized thylakoid proteins extracted from wild type and *npq1lut2 *membranes. WT and *npq1lut2 *rosettes were pre-treated for 48 h at 10°C and low light as described in methods, then were exposed to photoxidative conditions (1000 μmol photon m^-2 ^s^-1^, 10°C, 16 h light/8 h dark). Leaves were harvested and thylakoids isolated before stress (0) and at same time after 1, 2 and 5 days of HL.

The results in Figure 2 show that only SOSG fluorescence differed according to the genotype, with significantly higher fluorescence in mutant leaves (Figure 2C); there was no significant difference in the DCF and ProxF signals (Figures 2A, B). These results show that the accumulation of ^1^O_2 _is selectively enhanced in *npq1lut2 *mutant leaves whereas the other ROS are accumulated at the same level in both the mutant and wild-type. These data were confirmed by determining the extent of protein oxidation in thylakoids using the Millipore OxyBlot kit: *npq1lut2 *plants showed evidence of increased protein carbonylation after 1 day exposure to excess light, whereas wild-type plants took 5 days before an increase was detectable and the amplitude of the signal was far lower (Figure 2D).

It has been reported that the chloroplast can control the rate of transcription in the nucleus via the redox state of PQ [32], the ADP/ATP ratio and the redox state of stromal components [66,67]. In order to determine whether differences in gene expression between wild-type and mutant plants reflected differences in ^1^O_2 _steady-state accumulation, we studied the kinetics of these parameters under the same stress conditions described above. There were no major differences in qP, ascorbate and glutathione redox state, and ADP/ATP ratio, but there was a significantly greater reduction in maximum PSII photochemical efficiency (Fv/Fm) in mutant within the first 2 d, which reflects PSII damage induced by high ^1^O_2 _levels (Table 4).

**Table 4:** Time-course of main chloroplast parameters putatively involved in the regulation of gene expression, as previously reported [32,66,67].

	WT						*npq1lut2*					
	
Time (hours)	0	2	24	48	72	144	0	2	24	48	72	144
**qP**	1	0,20 ± 0,06	0,15 ± 0,07	0,03 ± 0,02	0,05 ± 0,01	0,10 ± 0,08	1	0,07 ± 0,03	0,08 ± 0,05	0,02 ± 0,01	0,07 ± 0,08	0,13 ± 0,08
**Fv/Fm**	0,79 ± 0,01	0,48 ± 0,07	0,42 ± 0,03	0,47 ± 0,07	0,43 ± 0,17	0,49 ± 0,07	0,79 ± 0,01	0,51 ± 0,13	0,22 ± 0,10	0,07 ± 0,03 *	0,45 ± 0,09	0,46 ± 0,13
**ADP/ATP**	2,2 ± 0,2	1,8 ± 0,1	2,2 ± 0,9	2,2 ± 0,6	2,1 ± 0,3	2,3 ± 0,4	2,1 ± 0,2	1,7 ± 0,1	2,5 ± 0,6	2,4 ± 0,1	1,8 ± 0,1	2,3 ± 0,1
**GSH/(GSH+GSSH)**	91,3 ± 9,5	96,2 ± 14,5	96,3 ± 7,1	95,1 ± 8,2	93,2 ± 10,4	85,7 ± 5,2	96,9 ± 8,5	92,1 ± 7,5	96,6 ± 3,0	91,6 ± 6,1	79,5 ± 20,4	78,5 ± 10,3
**Asc/(Asc+DHA)**	74,5 ± 4,1	73,1 ± 1,2	78,6 ± 2,4	75,6 ± 2,2	68,9 ± 4,0	71,2 ± 5,3	69,1 ± 2,2	67,8 ± 4,4	74,5 ± 3,4	74,2 ± 2,8	72,9 ± 2,9	53,2 ± 4,0 *

WT and *npq1lut2 *rosettes were pre-treated for 48 hrs at 10°C (see methods for details), then were exposed to photoxidative conditions (1000 μmol photon m^-2 ^s^-1^, 10°C, 16 h light/8 h dark). Leaves were harvested, then used for analysis of chlorophyll fluorescence parameters or immediately frozen in liquid nitrogen for measurements of metabolites, at the same time of the day over a 6-day-long stress period. Abbreviations: qP, photochemical quenching; Fv/Fm, maximal PSII photochemical efficiency; GSH/GSSG, glutathione reduced/oxidized; Asc, ascorbate; DHA, dehydroascorbate. Values that differ significantly between wild type and npq1lut2 mutant plants (Student's t-test, p < 0.02) are marked by an asterisk.

Nevertheless, acclimation to stress conditions led to the recovery of Fv/Fm in both wild-type and *npq1lut2 *plants within 3 days (Table 4). The levels of ascorbate and glutathione increased in both genotypes upon HL treatment. Ascorbate accumulates at even higher extent in wild-type leaves than *npq1lut2 *in response to HL. On the contrary, the total amount of ATP and ADP was only slightly affected by stress treatment in both genotypes (Additional file 2: Figure S3).

### Regulation of photosynthetic pigment metabolism

**Figure 3:**
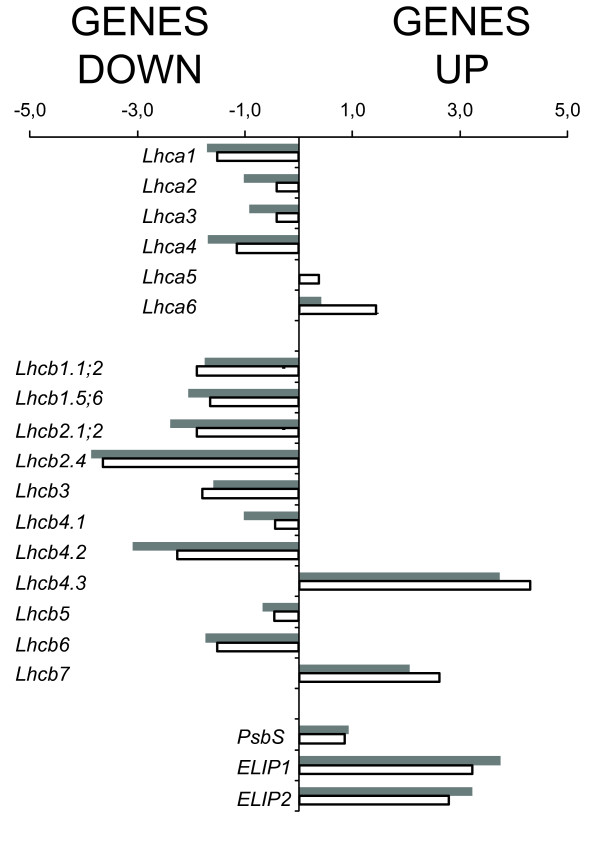
***Lhc *gene expression**. Light harvesting complex (*Lhc*) gene expression after 24 h stress (1000 μmol photons m^-2 ^s^-1^, 10°C) in wild-type (gray bars) and *npq1lut2 *(white bars) plants. For each sample, the average of three repetitions was used to calculate the fold change, which is expressed using the log_2 _scale. The genes significantly down-regulated after RMA analysis are *Lhca1 *(251814_at), *Lhcb1 *(255997_s_at; 267002_s_at), *Lhcb2 *(263345_s_at; 258239_at), *Lhcb3 *(248151_at), *Lhcb4.2 *(258993_at), *Lhcb6 *(259491_at). The genes significantly up-regulated after RMA analysis are *Lhcb4.3 *(265722_at), *Lhcb7 *(259970_at), *ELIP1 *(245306_at) and *ELIP2 *(258321_at). *Lhca4 *(252430_at) was significantly down-regulated only in the wild-type plants whereas *Lhca6 *(256015_at) was significantly up-regulated only in the mutant plants.

We next investigated the transcriptional regulation of genes in the chlorophyll and carotenoid metabolic pathways, since these pigments play an important role in light harvesting and photoprotection, and pigment-protein complexes are the main sources of ^1^O_2 _in thylakoids when the photosynthetic machinery is overexcited [46,68]. Specifically, we studied the carotenogenic genes (Additional file 1: Table S5) and the *Lhc *(Figure 3) and *Psa/Psb *gene families (Table 5) to see if their expression was sensitive to HL treatment.

**Table 5:** Photosystem II and photosystem I genes.

Locus identifier	Description	Fold Changes in WT	Fold Changes in *npq1lut2*
ATCG00680	CP47, subunit of PSII reaction centre	-0.9	-0.1
ATCG00020	D1, subunit of PSII reaction centre	0.3	0.5
ATCG00270	D2, subunit of PSII reaction centre	-0.1	0.5
ATCG00430	Photosystem II G protein	-0.8	0.1
ATCG00080	Photosystem II I protein	-1.0	-0.7
ATCG00070	Photosystem II K protein	-1.4 *	-0.9
AT4G05180	PSBQ2, oxygen-evolving enhancer protein 3	-1.9 *	-1.4 *

AT5G64040	PsaN	-1.2 *	-0.4
AT1G03130	PsaD	-2.0 *	-1.0 *
AT2G20260	PsaE	-1.7	-1.0
ATCG00350	PsaA	0.0	0.8
ATCG00340	PsaB	0.1	0.6
AT1G08380	PsaO	-1.6 *	-0.9
AT1G31330	PsaF	-1.0	-0.7

This table presents the subset of genes belonging to each photosystem core complex. The ratio between *npq1lut2 *and wild-type plants after 24 h stress is expressed as a log_2 _scale. Marked fields represent probe sets with a significant changes after RMA analyses.

**Figure 4:**
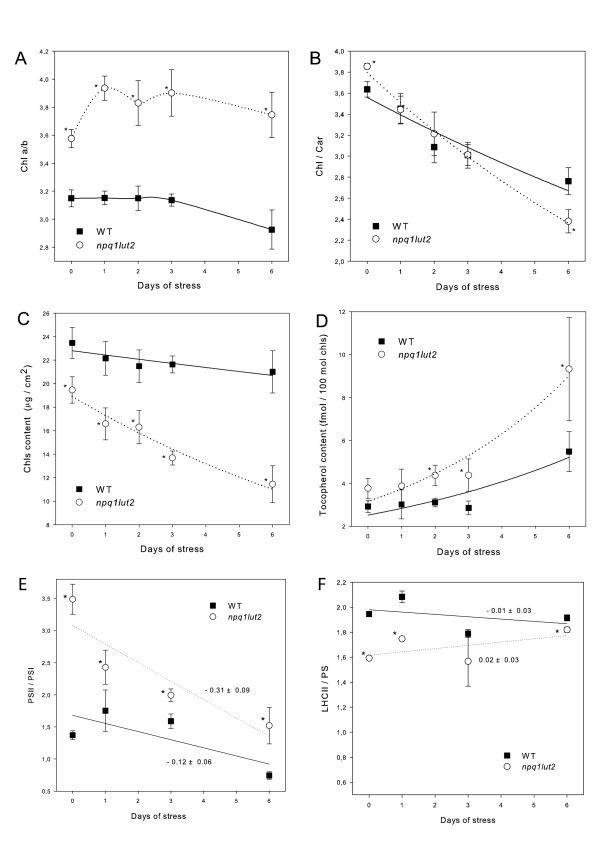
**Biochemical characterization of thylakoid membrane composition under high light stress**. Chlorophylls (A, C), carotenoids (B) and tocopherol (D) content of WT and *lut2npq1 *plants were measured on leaf acetone extracts as described in "Material and Methods". (E, F) Stoichiometry between photosynthetic pigment-binding complexes under high light stress. PSII/PSI ratio (E) and biochemical antenna size (LHCII/PS ratio, F) were determined by both non-denaturing Deriphat-PAGE and immunoblot-titration using specific antibodies (see "Material and Methods" for details). Symbols and error bars show respectively means ± SD.

We identified several genes in the chlorophyll biosynthetic pathway that were differentially regulated in wild-type and mutant plants exposed to excess light at low temperature. We found that heme oxygenase 3 (AT1G69720), which catalyzes the rate-limiting step in the degradation of heme, uroporphyrin III C-methyltransferase (AT5G40850), which is involved in siroheme biosynthesis, and glutamate-1-semialdehyde 2,1-aminomutase (AT3G48730) and uroporphyrinogen III synthase (AT2G26540), which catalyze steps in porphyrin and chlorophyll metabolism, were induced much more strongly in the mutant. In contrast, for a gene encoding protochlorophyllide reductase B (AT4G27440), which is involved in the light-dependent step of chlorophyllide *a *biosynthesis, was repressed specifically in the mutant (Additional file 1: Table S5). These results indicate that HL-treatment on *npq1lut2 *plants redirects the porphyrin biosynthetic pathway from chlorophyll synthesis to the production of heme and siroheme, thus reducing the total amount of chlorophyll in the overexcited system. Consistently, the chlorophyll content per leaf area decreased more rapidly in mutant plants than wild type plants exposed to excess light (Figure 4C).

Several genes in the xanthophyll biosynthesis pathway were up-regulated in both wild-type and mutant plants, with stronger induction after 24 h. These included phytoene synthase (AT5G17230), phytoene dehydrogenase (AT4G14210, AT1G57770), lycopene-β-cyclase (AT3G10230), β-carotene hydroxylase *chy1 *(AT4G25700) and zeaxanthin epoxidase (AT5G67030). The strong up-regulation of carotenogenic genes in response to elevated irradiation would sustain chloroplast acclimation. The carotenoid content of whole leaves supported this hypothesis, since mutant plants acclimated to a lower Chl/Car ratio than wild-type plants after 6 d exposed to excess light at low temperature (Figure 4B), suggesting that ^1^O_2 _signalling can account for the modulation of xanthophyll content in the thylakoid membrane. The differential expression of *VTE1 *in wild-type and mutant plants (Additional file 1: Table S6) is consistent with the higher tocopherol production in the mutant plants exposed to stress conditions (Figure 4D).

### Regulation of pigment-binding proteins

Lhc proteins are located within the thylakoid membranes, where they coordinate the chlorophylls and carotenoids. They are encoded by a superfamily of nuclear genes whose transcription [69], translation [70-72] and protein accumulation [20,35] are finely regulated in response to environmental cues. The expression profiles of most *Lhc *genes were very similar in wild-type and mutant plants exposed to excess light for 24 h (Figure 3). The genes significantly up-regulated in both genotypes were *Lhcb4.3 *(AT2G40100), *Lhcb7 *(AT1G76570), *ELIP1 *(AT3G22840) and *ELIP2 *(AT4G14690), indicating their involvement in the general stress response. However, *Lhca4 *(AT3G47470) was significantly down-regulated only in wild-type plants, whereas *Lhca6 *(AT1G19150) was up-regulated only in the mutant.

Furthermore, many genes encoding PSII and PSI core complex subunits were significantly down-regulated in wild-type plants exposed to excess light, but up-regulated or marginally down-regulated in the mutant, i.e. *CP47 *(ATCG00680), *D2 *(ATCG00270), *PsbG *(ATCG00430), *PsbI *(ATCG00080), *PsbK *(ATCG00070), *PsaD *(AT1G03130), *PsaO *(AT1G08380) and *PsaN *(AT5G64040). Table 5 shows the gene expression ratios on the log_2 _scale. Marked fields represent probe sets showing a significant change. CP47 was more strongly repressed in wild-type compared to mutant plants, with a similar tendency observed for other probe sets such as D2 and PsaA, for which down-regulation or no modulation was observed in wild-type plants while up-regulation was observed in the mutant. These finding indicate that the main response to excess light at low temperatures is a general repression of photosynthesis-related genes, but HL treatment in mutant leaves results in specific transcriptional re-programming of the core subunits of both photosystems, relieving the transcriptional repression in wild-type leaves. Biochemical analysis of thylakoid pigment-protein composition during stress treatment showed that the photosynthetic machinery acclimates by reducing the PSII/PSI ratio (Figure 4E), but there is little change in the antenna size as detected by the LHCII/PSII ratio (Figure 4F). These results agree with previous reports showing that when PSII becomes rate-limiting for photosynthetic electron transport, changes in photosystem stoichiometry occur to counteract this inefficiency [32]. Although the redox state of PQ is the same in both genotypes (Table 4), genes encoding PS core complexes are differentially expressed and there are differences in the rate at which the PSII/PSI ratio declines. The faster reduction in the PSII/PSI ratio in mutant leaves, independent of PQ redox state or PSII photoinhibition (Table 4), suggests a ROS-dependent signal transduction pathway that facilitates the acclimatory modulation of thylakoid composition.

### Chloroplast reorganization in response to ^1^O_2 _accumulation

**Figure 5:**
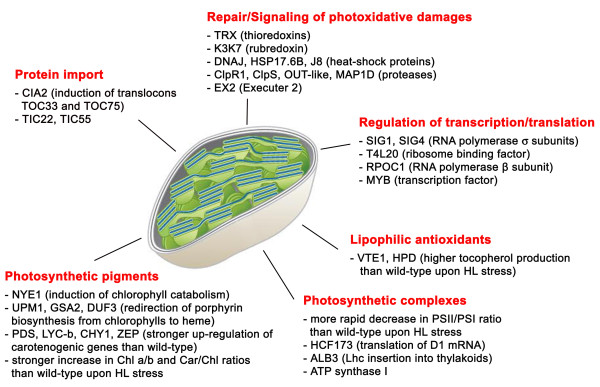
**Transcriptional induction, dose-dependent to ^1^O_2_, of genes encoding chloroplast proteins**. Most relevant genes encoding chloroplast proteins, that showed a statistically significant response to high light at low temperature only in *npq1lut2*, are reported. Up-regulation is defined as described in Methods. Abbreviations: ALB, albino; CIA, chloroplast import apparatus; CHY, carotene hyroxylase; DUF, uroporphyrinogen III synthase; GSA, glutamate-1-semialdehyde 2,1-aminomutase; HCF, high chlorophyll fluorescence; HPD, 4-hydroxyphenylpyruvate dioxygenase; LCY, lycopene cyclase; NYE, non-yellowing; PDS, phytoene desaturase; SIG, RNA polymerase sigma subunit; TIC/TOC, translocon of inner/outer chloroplastic membrane; UPM, urophorphyrin methylase; VTE, tocopherol cyclase; ZE, zeaxanthin epoxidase.

Several signals are thought to pass from the plastid, either directly or indirectly, through the cytoplasm to the nucleus, where they modulate gene expression under stress [25]. After acetonic extraction, pigment analysis showed that the chlorophyll a/b ratio was higher in the mutant than the wild-type and this difference increased under stress (Figure 4A), reflecting the changing PSII/PSI ratio in the mutant upon HL treatment (Figure 4E) rather than a reduction in antenna size (Figure 4F). Under stress, *Lhc *transcription was inhibited to the same extent in both genotypes, whereas photosystem core genes were down-regulated more strongly in the wild-type plants. This is consistent with the significant increase in the Chl a/b ratio observed in the mutant, but there was no modulation of *Ftsh *expression to explain the more rapid degradation of pigment-protein complexes (Additional file 1: Table S6). The Chl/Car ratio differs significantly between the two genotypes, with wild-type plants showing a 24% reduction under stress, and mutants showing a 38% reduction (Figure 4B). Evidence for oxidative stress was found in the pattern of antioxidant compounds, e.g. glutathione S-transferase, methionine sulfoxide reductase and tocopherol (Additional file 1: Table S6). Several genes showing induction in *npq1lut2 *only encoded chloroplast proteins, that might be involved in cell protection against the damaging effect of ROS (Figure 5). Since most were induced after 24 h in the mutant, it suggests that induction occurs only when ^1^O_2 _accumulation exceeds a threshold level (Additional file 1: Table S7).

## Discussion

We have carried out a comparative analysis of wild-type Arabidopsis plants and the double mutant *npq1lut2 *in terms of mRNA levels, metabolite levels and physiological functions in response to conditions leading to oxidative stress. The *npq1lut2 *xanthophyll biosynthesis mutant was used to study the effect of ^1^O_2 _accumulation on physiological stress responses [47,49]. This mutant lacks violaxanthin de-epoxidase (NPQ1) and lycopene-ε-cyclase (LUT2) activities, and is susceptible to photooxidative stress when exposed to excess light at low temperatures [47]. Under normal growth conditions the gene expression profile of the mutant is almost identical to that of wild-type plants, but differences become evident following exposure to excess light (1000 μmol m^-2 ^s^-1^) at low temperature (10°C). At time 0 (before stress), 18 genes were down-regulated in the mutant relative to wild-type plants, although the expression of those genes could be directly or indirectly regulated by the absence of lutein and zeaxanthin. Also, during high light treatments lutein and zeaxanthin could play a signalling role, directly or by compounds derived from them. The effect of individual carotenoids on transcription has not been analyzed in detail, but it is clear that the carotenoid content of the chloroplast affects gene expression under both normal and stress conditions, and affects chloroplast to nucleus communication [13,73,74]. Here, we show that ^1^O_2 _accumulation in response to excess illumination within the physiological range is perceived as a signal to regulate significant number of nuclear genes encoding chloroplast proteins, facilitating acclimation to stress, but is not sufficient to induce apoptosis.

### Xanthophyll mutants are valuable for the analysis of ^1^O_2 _signalling

The suitability of the *lut2npq1 *mutant for the analysis of ^1^O_2 _signaling was confirmed by comparing physiological parameters and ROS accumulation in relation to wild-type plants. Previous results [47,75,76] showed that *lut2 *mutation in Arabidopsis only affected few physiological parameter (increase in PSII/PSI and Chl a/b ratios, reduced efficiency of state transitions and LHCII trimerization); however, photosynthetic efficiency and growth rate in *lut2 *plants were indistinguishable from wild-type. We cannot exclude that differences between the two genotypes at the onset of HL treatment could be responsible of some of the differential responses at transcriptome level. However, WT and *npq1lut2 *accumulate different amounts of ^1^O_2 _from their chloroplasts before stress treatment (Figure 2, T = 0) as further confirmed by transcript levels at time 0 showing no major differences in gene regulation between WT vs mutant. Therefore, if a differential ^1^O_2 _accumulation occurs even in low light, it is below the threshold level that makes ^1^O_2 _a signal in the regulation of gene expression.

Present results demonstrate that ^1^O_2 _is the only ROS differentially accumulated in the mutant with respect to WT upon HL treatment, while this mutations does not differentially affect the main parameters that, until now, have been related to gene expression regulation in HL. Indeed, following illumination at 1000 μmol m^-2 ^s^-1 ^and 10°C, the photosynthetic electron transport chain was reduced to the same extent in both genotypes (Table 4). This allowed us to monitor the impact of excess light on the redox state of the PQ pool, a physiological parameter that has been proposed to have a specific role in chloroplast to nucleus signalling during stress acclimation [32]; therefore, the differential gene expression in wild-type *vs *mutant plants cannot be attributed to changes in the PQ redox state, confirming data from previous studies [35]. Additional proposed signalling molecules, such as reduced forms of ROS, the redox state of the stoma redox component (GSH/GSSG, Asc/Asc+DHA), and the ATP/ADP ratio [67] were indistinguishable in the two genotypes (Table 4 and Additional file 2: Figure S3), suggesting they are not major transcriptional regulators in response to photo-oxidative stress conditions used in this report. Therefore, all data presented suggest that gene expression changes described could be reasonably ascribed to singlet oxygen, even if we cannot exclude that other factors could act as signal in *npq1lut2 *plants, together with singlet oxygen, in the modulation of gene expression.

The *npq1lut2 *mutant shows a selective loss of lutein, which is active in ^3^Chl* quenching [47], and of zeaxanthin, which is an ^1^O_2 _scavenger [47,48,77,78], therefore the mutant specifically accumulates ^1^O_2 _but not other ROS (Figure 2C) [47,79]. It should be noted that the change in xanthophyll composition marginally affects the composition of the photosynthetic apparatus in the mutant [47] while photosynthetic electron transport and growth rate are the same in both genotypes, therefore ^1^O_2 _steady-state accumulation in the *npq1lut2 *mutant occurs only in response to excess light conditions (Figure 1 and Additional file 1: Table S4). Thus, *npq1lut2 *compares favourably with the *flu *mutant [29] in which ^1^O_2 _is produced through the accumulation of Chl biosynthesis precursors, eventually leading to complete chloroplast bleaching. The present study on *npq1lut2 *is the first case in which ROS generation has been elicited in its natural site (i.e. within thylakoid membranes) rather than provided from outside or produced by photosensitizing metabolic precursors soluble in the chloroplast stroma. The level of PSII photoinhibition we found in *npq1lut2 *is not dramatic, since the photochemical efficiency of the mutant starts to acclimate to the stressing conditions after 4 days of HL (Table 4). In the *flu *mutant, over-accumulation of the photosensitizer Pchlide results in a stronger photosensitive phenotype, with extensive cell death as early as 1 h after the onset of illumination, and visible necrotic lesions formed 2 to 3 h later. Clearly, the level of stress applied in our experiment is far lower from that described in (Op den Camp et al. Plant Cell 2003) and is followed by a successful acclimative response as in a physiological response. Therefore we strongly support the notion that in our experimental conditions, ^1^O_2 _acts primarily as a signal that modulates chloroplast acclimation to photoxidative stress.

The photosynthetic parameters and metabolic indicators discussed above (i.e. F_v_/F_m_, Chl a/b and Chl/Car ratios, PSI/PSII ratio) show that the chloroplast function and communication between the chloroplast and cytoplasm are impaired in the mutant, while the differential expression of nuclear genes encoding chloroplast proteins confirms that the chloroplast is a central switch of the plant's response to cold and light stress [13,74]. We can now decipher the contribution of ^1^O_2 _signalling to the stress acclimation response. A similar system was previously used with the mutant *npq1lor1 *of the green alga *Chlamydomonas reinhardtii*. Nevertheless, in Arabidopsis we identified a fast component of gene expression regulation by ^1^O_2 _at 2h that was not detected in Chlamydomonas [80].

### The npq1lut2 transcriptome integrates the ROS signalling network

Oxidative stress is a complex process that can be triggered by a range of environmental, biotic and developmental factors. It is therefore not surprising that different pathways can be induced, depending on the nature of the stress. Previous studies using a catalase-deficient mutant exposed to excess light identified genes that are differentially expressed in response to H_2_O_2 _accumulation, leading to the discovery that H_2_O_2 _regulates anthocyanin biosynthesis [28]. Several reports have also proposed that ^1^O_2 _has a signalling role [81,82].

Here we have determined the photoprotective effect of two xanthophylls when plants are exposed to excess light at low temperatures. Only 18 genes were found to be differentially expressed between wild type plants and the *npq1lut2 *mutant under normal conditions, probably reflecting the absence of lutein and zeaxanthin in the mutant (Additional file 1: Table S4). However, when the plants were exposed to excess light at a low temperature, a group of 67 genes encoding chloroplast proteins was specifically repressed in wild type plants, whereas the same genes were not affected in the mutant. This is intriguing because a nuclear mutation affecting chloroplast xanthophyll composition is clearly able to regulate gene expression and ultimately chloroplast acclimation. We can thus conclude that the expression of some nuclear genes depends on the xanthophyll content directly or indirectly, via its impact on ^1^O_2 _accumulation (Figure 2C). We do not exclude that lutein, zeaxanthin and products of their metabolisms play a signalling role under stress. Indeed, carotenoids can play a clear signalling role [83]. Here we want to highlight the correlation between gene expression regulation and ^1^O_2 _steady-state accumulation in a mutant lacking two photoprotective xanthophylls. One possibility is that a subset of genes in Table 2 responds to the change in ^1^O_2 _accumulation within the thylakoid membranes, e.g. those encoding glutaredoxin (AT1G03850), ATP-dependent protease La (AT1G75460), DNAJ heat shock N-terminal (AT4G13830) and enzymes involved in phylloquinone and plastoquinone biosynthesis (AT1G60600). Functional annotation of the 38 uncharacterized genes in this list will help further to decipher how gene regulation by lutein and zeaxanthin occurs under oxidative stress, as shown in previous studies [84,85].

One group of genes specifically modulated in the *npq1lut2 *mutant overlaps with those regulated in *flu *(Additional file 1: Table S8), a reference mutant used in the study of ^1^O_2 _signals [50,86] in agreement with the high level of ^1^O_2 _accumulation measured in *npq1lut2 *(Figure 2C). Also in the attempt of comparing the response in *npq1lut1 *vs *flu*, we performed a more sophisticated statistical analysis comparing *npq1lut2 *transcriptome and *flu*/*executer *transcriptome [53]. The conditions used in the two experiments are different as demonstrated by the high number of genes (2420 probe-sets) differentially expressed in the two wild-types (Additional file 2: Figure S4A). A low level of overlap between *npq1lut2 *and *flu*/*executer *transcript response was detectable (Additional file 2: Figure S4B) showing that transcriptomic analysis performed in different labs under different experimental conditions must be compared with precaution as shown by previous papers [7,36]. Comparative transcriptomic analysis of the ^1^O_2 _response signature showed that the cluster of genes regulated by ^1^O_2 _in both *flu *and *npq1lut2 *is not modulated in all oxidative stress cases analyzed to date. However, we identified a subset of genes affected by ^1^O_2 _and O_3_, whereas there is negative correlation between the genes modulated by ^1^O_2 _and those modulated by H_2_O_2 _(Table 6). This antagonistic transcriptional regulation mediated by ^1^O_2 _and H_2_O_2 _supports previous data showing cross-talk and antagonistic H_2_O_2 _and ^1^O_2 _signalling in *flu *mutants under stress overexpressing the thylakoid-bound ascorbate peroxidase [30]. The molecular basis of these opposing responses appears to reflect the presence of specific *cis*-regulatory elements responsive to either ^1^O_2 _or H_2_O_2 _within the corresponding promoters [87]. A new and close relationship among ROS was recently demonstrated, where each ROS species activates a specific response, but the pathways converge to produce a clear ^1^O_2 _signature in lipid peroxidation [52].

**Table 6:** Expression of genes up- and down-regulated in different ROS accumulating conditions.

UP regulated											
Probeset	Locus identifier	*flu*	*n1l2*	Ozono	MV 2 h	MV 4 h	*vte2*	*vte1*	*cat*	DCMU	Description
253259_at	At4g34410	**6,60**	**1,29**	**2,17**	**1,21**	-0,24	-1,13	**0,20**	-2,93	-0,62	RRTF1, AP2 domain-containing transcription factor
253832_at	At4g27654	**6,23**	**1,02**	**0,77**	**0,53**	**1,53**	**0,88**	**1,29**	-3,59		unknown protein
248793_at	At5g47240	**5,78**	**1,17**	**2,21**	**0,35**	**1,04**	**0,20**	-0,11	-2,20		ATNUDT8, Nudix hydrolase homolog 8
247360_at	At5g63450	**5,51**	**1,21**	**1,47**	**2,84**	**1,08**	**0,19**	-0,80	-0,81		CYP94B1, oxygen binding cytochrome P450
266821_at	At2g44840	**5,40**	**1,03**	**2,60**	**2,75**	**1,79**	**0,28**	**0,36**	-2,74	-0,40	Ethyne responsive element binding factor 13
262354_at	At1g64200	**4,82**	**2,21**	**1,36**	-0,10	**0,23**	-0,02	-0,50	**0,03**		Vacuolar H+-ATPase subunit 3
247030_at	At5g67210	**4,62**	**1,22**	**1,19**	-0,33	**0,11**	-0,10	-2,48	-1,03		nucleic acid binding/putative ribonuclease
256021_at	At1g58270	**3,92**	**4,23**	**0,36**	**0,30**	-0,60	**0,41**	-0,18	-0,80		ZW9
266977_at	At2g39420	**3,66**	**1,45**	**1,63**	**0,26**	-0,05	-0,10	**0,27**	-0,75	-0,34	esterase/lipase/thioesterase family protein
255941_at	At1g20350	**3,33**	**1,46**	**4,30**	**0,30**	-0,18	-2,10	**1,05**	**1,08**		TIM17, mitochondrial inner membrane translocase
263320_at	At2g47180	**3,10**	**1,10**	**1,71**	-0,24	**0,82**	**2,04**	**0,99**	-0,36		AtGOLS1 Galactinol Synthase 1
266418_at	At2g38750	**2,47**	**1,31**	**0,80**	-0,42	-0,69	**1,04**	-1,97	**0,09**	-0,43	ANNAT4, Annexin *Arabidopsis *4; calcium ion binding
264986_at	At1g27130	**2,46**	**1,07**	**1,52**	-0,08	**0,72**	-0,21	-0,36	**0,52**		ATGSTU13, glutathione S-transferase 13

											

**DOWN regulated**											
**Probeset**	**Locus identifier**	***flu***	***n1l2***	**Ozono**	**MV 2 h**	**MV 4 h**	***vte2***	***vte1***	***cat***	**DCMU**	**Description**

247638_at	At5g60490	-2,44	-0,92	**0,01**	-0,22	**0,17**	**0,30**	**0,60**	**0,17**		FLA12__FLA12 (fasciclin-like arabinogalactan-protein 12)
252573_at	At3g45260	-2,38	-0,37	**0,19**	**0,73**	**0,37**	-0,43	**0,13**	**0,05**		zinc finger (C2H2 type) family protein
258370_at	At3g14395	-2,46	-0,69	**0,07**	-2,76	-0,01	-0,03	**1,29**	**0,47**		unknown protein
255149_at	At4g08150	-2,85	-0,97	-0,23	-1,98	**1,33**	**0,25**	-0,10	-0,38		KNAT1_BP__KNAT1 (BREVIPEDICELLUS 1); transcription factor
259903_at	At1g74160	-2,34	-0,72	**0,26**	-0,26	**0,16**	**0,05**	**0,25**	-0,58		unknown protein
262783_at	At1g10850	-2,47	-0,37	-0,27	**0,07**	**0,41**	-0,21	-0,67	-0,10		ATP binding/protein serine/threonine kinase
261883_at	At1g80870	-2,43	-0,48	-0,55	-0,36	-0,01	**0,84**	**0,85**	**0,44**		protein kinase family protein
247463_at	At5g62210	-2,77	-0,91	-1,24	**1,16**	-0,22	**0,36**	-1,72	-1,66		embryo-specific protein-related
252746_at	At3g43190	-3,53	-0,51	**0,19**	**0,89**	-0,40	-0,31	-1,83	-0,10		SUS4__SUS4; UDP-glycosyltransferase/sucrose synthase
250891_at	At5g04530	-2,64	-0,47	-0,35	-0,52	**0,27**	-0,10	-0,22	-1,34		beta-ketoacyl-CoA synthase family protein
260693_at	At1g32450	-2,51	-0,26	-0,23	-0,21	**0,22**	-0,85	-0,70	-0,50		proton-dependent oligopeptide transport (POT) family protein
250344_at	At5g11930	-2,82	-0,97	**0,14**	-0,39	-1,01	-0,45	**0,38**	-1,17		glutaredoxin family protein

The transcription regulation of genes specifically responding to ^1^O_2 _in *flu *and *npq1lut2 *mutants was compared to various experiments by using mutants and/or treatments. The ratio between treated and control plants is expressed as a log_2 _scale. For each sample, the average of three repetitions was considered.

The genome-wide hypersensitive response is more strongly induced in *flu *mutants than in *npq1lut2 *mutants (Additional file 1: Table S9). Among 369 genes significantly up-regulated following infection with *Pseudomonas *DC3000 (*avrRpm1*) [88], 292 were also detected in the *flu *and *npq1lut2 *transcriptomes with 267 induced in *flu *and only 69 in *npq1lut2 *(resulting in a far less pronounced apoptotic response). In agreement with this, we did not observe cell death in Arabidopsis plants by vital staining and DNA fragmentation analysis (data not shown). Because *npq1lut2 *specifically showed higher ^1^O_2 _steady-state accumulation (Figure 2), this implies that cell death is not a specific or immediate response to ^1^O_2 _in the absence of the most effective photoprotection mechanisms present in wild-type plants, at least under our experimental conditions. However, we cannot exclude the possibility that higher levels of ^1^O_2 _accumulated under non-physiological conditions, might induce cell death.

Recent work by Apel and co-workers revealed that *EXECUTER *genes are involved in the early response to ^1^O_2 _in Arabidopsis by the transduction of ^1^O_2 _signals from the chloroplast to the nucleus in the *flu *mutant [51,53]. ^1^O_2 _accumulation in *npq1lut2 *induced the expression of *ex2 *but not *ex1*, but there was no effect in similarly-treated wild-type plants, confirming that ^1^O_2 _oxygen signals are measurable in the *npq1lut2 *transcriptome and that *EX1 *and *EX2 *might respond differently to environmental cues.

### Xanthophylls modulate the pigment composition of thylakoid membranes

It is well documented that plants acclimate to different light conditions by regulating their carotenoid composition [89]. It is worth noting that the higher rate of ^1^O_2 _accumulation in *npq1lut2 *plants corresponds to the induction of genes representing the β-β branch of carotenoid biosynthesis (β-carotene hydroxylase, zeaxanthin epoxidase, lycopene β-cyclase; Table S5). In thylakoid membranes, the accumulation of β-β xanthophylls would increase the ability of plants to synthesize zeaxanthin and neoxanthin when needed, thus facilitating the response to excess light. Indeed, these β-β xanthophylls have both an important role in photoprotection [90-92] and mutants lacking such compounds undergo irreversible photo-oxidation when exposed to excess light [90]. Growth under intense light caused carotenoid levels to increase in *npq1lut2 *plants compared to the wild-type (Figure 4B), and because carotenoids scavenge ^1^O_2 _or directly quench ^3^Chl*, the increased Car/Chl ratio appears to be a protective mechanism [20,93].

Besides carotenoids, plants synthesize other antioxidants such as tocopherol (vitamin E). This lipophylic compound is localized exclusively in the lipid phase of the thylakoid membranes, and is an active ^1^O_2 _scavenger [10,94]. Higher levels of tocopherol were observed in the leaves of *npq1 *mutant plants after 3 d of excess light stress [95], and it was proposed to have a primary role in the prevention of lipid peroxidation promoted by ^1^O_2_. We found that *npq1lut2 *plants under chilling stress accumulated tocopherols to higher levels than wild-type plants when exposed to excess light for 6 d (Figure 4D) and contained ~70% more α-tocopherol. Tocopherol synthesis is therefore strongly induced by excess light in the mutant, particularly given the rapid consumption due to the increased rate of ROS accumulation. The biochemical analysis was consistent with the transcriptomic data, showing stronger and faster induction of tocopherol synthesis genes in the mutant, e.g. HPD (AT1G06570) and VTE1 (AT4G32770) (Additional file 1: Table S6).

Tetrapyrrole synthesis must also be regulated under excess light stress to prevent damage to the photosynthetic machinery, and when photo-oxidative stress accelerates the degradation of pigment-protein complexes, the synthesis of chlorophyll must slow down to compensate. We therefore measured changes in the total Chl content as well as in Chl a/b ratio. The tetrapyrrole pathway is regulated by metabolic intermediates at the transcriptional and post-translational levels [96]. In particular, heme is a well known repressor of early steps in the Chl synthesis pathway [97]. Crosstalk between tetrapyrrole biosynthesis and ^1^O_2 _was demonstrated in *flu *mutants [98]. Our data clearly show that the higher levels of ^1^O_2 _accumulation in *npq1lut2 *mutants promote the expression of heme oxygenase 3 (AT1G69720) and uroporphyrin III C-methyltransferase (AT2G26540), resulting in higher levels of heme in mutant compared to wild-type plants (Additional file 1: Table S5). Furthermore, repression of protochlorophyllide reductase B (AT4G27440) in *npq1lut2 *could limit chlorophyll production, helping to reduce the number of pigment-protein complexes in the cell during chloroplast acclimation to excess light.

^1^O_2 _therefore appears to participate in a fine-tuning system that modulates chlorophyll biosynthesis and the accumulation of carotenoids and lipophylic antioxidant compounds in excess light stress, thereby increasing plant fitness under normal illumination.

### Xanthophylls affect the composition of the photosynthetic apparatus during acclimation

Light-harvesting complexes respond rapidly to changes in environmental conditions [99]. We showed that most *Lhc *genes have similar expression profiles in wild type and *npq1lut2 *mutant plants, even though they encode proteins that bind lutein and zeaxanthin. Many *Lhc *genes were down-regulated, with *Lhcb2.4 *the most strongly repressed (Figure 3). The exceptions were *Lhcb4.3*, *Lhcb7*, *PsbS *and *ELIPs*, consistent with data showing that the four corresponding antenna proteins participate in photoprotection [69,100,101].

Interestingly, the different isoforms of Lhcb4 (CP29) were modulated in distinct ways despite their very similar polypeptide sequence. In particular, although Lhcb4.1 and Lhcb4.2 were down-regulated in both genotypes under stress conditions, Lhcb4.3 [102] was induced in both genotypes to the same extent. This is consistent with previous studies showing the evolutionary conservation of genetic redundancy in the *Lhc *superfamily [103], and it suggests that different CP29 polypeptides may play significant and specific roles in acclimation.

Our expression data also suggested that several signals intersect to regulate the *Lhc *superfamily and that transcriptional regulation is only one component of a more complex process. The most striking change in thylakoid composition under stress was the progressive reduction in the PSII/PSI ratio, which was more pronounced in *npq1lut2 *mutants (Table 5 and Figure 5). Such a reduction may be necessary to prevent the over-reduction of photosynthetic electron chains [20] and likely reflects changes to the rates at which the various substrates are synthesized and destroyed. PSII destruction is higher in the mutant because of the excessive photo-oxidation, and we have provided evidence that genes encoding several PSII core complex subunits (and to a lesser extent those in the PSI core complex) are induced in the mutant and repressed in wild-type plants (Table 5). It is well known that the transcription and the translation of PSII and PSI genes is extremely complex and often uncoupled. Analysis of the barley PSI-less *viridis zb63 *mutant showed an over-reduction of PQ pool and an increase in PSII core content into thylakoid with respect to WT (Frigerio 2007); all these changes in PSII content occurs without changes in PsaA mRNA levels. Furthermore, in the *viridis zb63 *mutant, despite the absence of fully assembled PSI complex and the missed accumulation of any core polypeptides, all genes encoding PSI subunits are substantially expressed at the same level with respect to wild-type plants. These evidences suggest that a) regulation of photosystems accumulation could not only involve chronic PQ reduction [32] and b) regulation of composition of photosynthetic components could be mainly at the level of protein turn-over.

In contrast to previous reports [20], the loss of PSII content was not accompanied by a dramatic loss of bulk LHCII, probably because more time might be needed to achieve a functional antenna size final state under our growth conditions. Finally, ^1^O_2 _induces chloroplast ATP synthase protein I (AT2G31040) specifically in *npq1lut2 *mutants after 24 h exposure to excess light, and a higher level of ATP synthase was previously identified as one of the long-term responses that facilitate chloroplast acclimation to intense light [104].

### Chloroplasts respond to the accumulation of ^1^O_2 _by functional reorganization

We found that several genes showing dose-dependent induction by ^1^O_2 _encoded chloroplast proteins whose function is to protect cells against the damaging effect of ROS. Most were induced after 24 h specifically in the mutant, suggesting induction occurs only when ^1^O_2 _accumulation exceeds a threshold level (Additional file 1: Table S7).

Many of these proteins were thioredoxins, ^1^O_2_-quenching proteins that respond to oxidative stress [105]. This is consistent with previous reports showing that thioredoxins are protective proteins that maintain the cellular redox environment [106]. Others are involved in chlorophyll catabolism (At4g22920 and At5g13800), and their induction correlates with both the down-regulation of genes involved in tetrapyrrole biosynthesis (Table S5) and the accelerated reduction of chlorophyll levels in mutant leaves under excess light stress compared to similarly-treated wild type plants. Others encode heat shock proteins (Hsps-p23like, sHsps, DNAJ, J8) and proteases (Clp serine-type endopeptidase, ATP-dependent Clp protease, OUT-like cysteine protease, MAP1D Met-aminopeptidase), which function as molecular chaperones that suppress aggregation of proteins damaged by ROS, or to facilitate protein turnover (Table S7). Others are involved in either the synthesis or membrane-insertion of photosynthetic subunits, e.g. Hcf173 (At1g16720) is part of a thylakoid complex essential for the translation of *psb*A mRNA (encoding D1), and its induction in a mutant in which higher ^1^O_2 _accumulation increases the rate of D1 turnover is consistent, and *Alb3 *(At2g28800) has a role in the insertion of a subset of light-harvesting complexes into thylakoids (Table S7). The induction of a lipase (At5g11650) and FAD7 (fatty acid desaturase 7, At3g11170) facilitates the production of jasmonic acid, an elicitor released by chloroplast membranes under photo-oxidative stress. *EXECUTER2*, whose role in coupling ^1^O_2 _signalling from the chloroplast to nucleus has been described [53], was also up-regulated specifically in mutant plants.

The up-regulation of *CIA2 *(At5g57180) in the mutant after 2 and 24 h of excess light stress is particularly interesting because CIA2 is a transcription factor that specifically promotes the expression of genes encoding the translocon proteins Toc33 and Toc75, which are necessary for protein import into the chloroplast, and chloroplast ribosomal proteins [107]. In addition, both Tic22 (At5g62650) and Tic55 (At2g24820) were up-regulated in the mutant, and these encode components of the translocon on the chloroplast inner envelope membrane. Taken together, these data suggest that ^1^O_2 _plays a key role in fulfilling the increased demand for protein import into the chloroplast during photo-oxidative stress, reflecting the higher rate of protein damage and turnover, by co-ordinately up-regulating both protein import and translation [107].

## Conclusions

Xanthophylls accumulated within thylakoid membranes are compounds that participate actively to ROS scavenging and to the prevention of ROS synthesis. Our data provide evidences that xanthophylls modulate ^1^O_2_-dependent signals during the acclimation to high-light and low-temperature conditions. Indeed, in *npq1lut2 *double mutant ^1^O_2 _signalling facilitates the early fine-tuning of the expression of a group of genes encoding chloroplast proteins. This regulation does not correlate with the redox state of the PQ pool. Chloroplasts respond to these signals by a significant change in composition, resulting in rapid morphological and functional modifications. The response to ^1^O_2 _does not include cell death, even in the highly photosensitive *npq1lut2 *mutant.

## Methods

### Plant material and growth conditions

*Arabidopsis thaliana *plants, wild-type and T-DNA insertion mutants (*Columbia *ecotype) *npq1 *(At1G44446) and *lut2 *(At5G57030) were obtained from NASC collections [108]. Mutant *npq1lut2 *was obtained by crossing single mutant plants and selecting progeny by pigment analysis [47]. Plants were grown in pots filled with homogenous non-enriched compost and watered weekly with Coïc-Lesaint nutrient solution [109]. They were grown in a growth chamber for 6 weeks under controlled conditions (~120 μmol photons m^-2 ^s^-1^, 24°C, 8 h light/16 h dark, 70% relative humidity).

### Micorarray experiments and statistical analysis of data

Before transcriptomic analysis, 6 weeks old plants were transferred from controlled conditions above described to a cold chamber (10°C) under low-light conditions (25 μmol photons m^-2 ^s^-1^, continuous light) and maintained in this environment for 48 h in order to reduce the effect of the circadian clock [110]. Wild-type and *npq1lut2 *plants were then exposed to intense light (1000 μmol photon m^-2 ^s^-1^) using 150 W halogen lamps (Focus 3, Prisma, Verona, Italy) at 10°C. Samples for transcriptome analysis were collected at 0, 2 and 24 h of excess light treatment, and rapidly frozen in liquid nitrogen prior to RNA extraction.

Three biological replicates per treatment were analyzed by using the Affymetrix GeneChip^® ^Arabidopsis ATH1 Genome Array, which contains more than 22,500 probe sets representing 24,000 gene-specific tags (about 80 are chloroplast genes). For each biological repetition, RNA samples for a condition/genotype were obtained by extracting RNA from the entire rosette of eight pooled plants. Total RNA was quantified and then adjusted to a final concentration of 1 μg/μl. RNA integrity was assessed using the Agilent RNA 6000 nano kit and Agilent Bioanalyzer 2100 (Agilent Technologies, Palo Alto, CA). RNA samples were processed following the Affymetrix GeneChip Expression Analysis Technical Manual (Affymetrix, Inc., Santa Clara, CA). Scanned images were analyzed using the Gene Chip Operating Software v1.4. Expression analysis was carried out using default values. Quality control values, present calls, background, noise, scaling factor, spike controls, and the 3'/5' ratios of glyceraldehyde-3-phosphate dehydrogenase (AT3G04120) and actin (AT5G09810) showed minimal variation between samples. Raw data files (CEL files) were background-adjusted and normalized, and gene expression values were calculated using the Robust Multichip Analysis (RMA) [111] algorithm implemented in the statistical package R2.3.1 (R foundation) with the dedicated "Affy" library [112].

The "Affy" library was used to run the MAS 5.0 algorithm on raw data to produce a detection call for each probe set. Because non-expressed genes ("absent") represent experimental noise and can generate false positives, all the probe sets failing to show three "present calls" in at least one sample were removed from the analysis. Normalized data were imported into the GenespringGX7.3.1 (Agilent Technologies, Santa Clara CA) software for analysis. Each gene was normalized to the median of the measurements.

To identify differentially expressed probe sets, we applied a Welch t-test with Benjamini and Hochberg false discovery rate correction for multiple tests [113]. Differences in gene expression were considered to be significant when p < 0.05 and the ratio of expression levels was at least two-fold [114]. Clusters of genes with distinctive expression patterns were searched applying two algorithms: k-means [115] and QT (Quality Threshold) cluster analysis [116]. QT clustering algorithm groups genes into high quality clusters based on two parameters: "minimum cluster size" and "minimum correlation". The minimum cluster size was set to 10 and minimum correlation to 0.75 (Pearson correlation). To determine if certain classes of genes were over-represented within selected clusters of genes compared to the functional categories on the entire array, the MIPS *Arabidopsis thaliana *database (MatDB) (mips.gsf.de/projects/funcat) was employed [54].

Data from other experiments were obtained as additional data from published papers [36,85] or downloaded from the European Bioinformatics Institute [117]. For published microarray data comparing a test sample and a control sample, genes were considered to be differentially expressed when they showed a log_2 _ratio of either ≥1 or ≤-1 [7].

### Quantitative real-time PCR (qRT-PCR)

Miroarray data were independently verified by qRT-PCR, using 3 μg total RNA from each sample. The RNA was reverse transcribed using an oligo(dT)18 primer with MoMLV Reverse Transcription Reagents (Promega) according to the manufacturer's standard protocol. The reaction was incubated at 40°C for 10 min, then 45°C for 50 min, and then at 70°C for 15 min to inactivate the reverse transcriptase. The cDNA was quantified using a QbitTM fluorometer (Invitrogen), diluted and used for q-PCR amplifications with specific primers.

Each qRT-PCR was performed with SYBR Green fluorescence detection in a qPCR thermal cycler (ABI PRISM 7300, Applied Biosystems). Each reaction was prepared using 5 μl from a 0.2 ng/mL dilution of cDNA derived from the reverse transcription, 10 μl of SYBR Green PCR Master Mix (Applied Biosystems), and 0.5 μM forward and reverse primers in a total volume of 25 μl. The cycling conditions were: 10 min at 95°C, followed by 40 cycles of 95°C for 15 s and 60°C for 1 min. Melting curve analysis was performed to identify non-specific PCR products and primer dimers.

Primers were designed using Primer Express^® ^Software for Real-Time PCR 3.0 (Applied Biosystems). Microarray data were validated by analyzing the expression profile at 0, 2 and 24 h excess light stress. The fold change between treated and untreated samples was compared to the transcriptomic data, and a linear correlation coefficient was calculated for each gene. The detailed qRT-PCR results for eight genes are shown in Additional file 2: Figure S2. Among 20 genes, 16 showed good correlation between qRT-PCR and microarray data (R^2 ^> 0.9).

### ROS measurements

steady-state accumulation of ROS in leaves was quantified using specific fluorogenic probes: singlet oxygen sensor green (SOSG), dichlorofluorescein (DCF) and proxyl-fluorescammine (proxF) (Molecular Probe, Eugene). SOSG is highly selective for ^1^O_2_, whose presence increases its 530 nm emission band [118]. DCF reacts with hydrogen peroxide (H_2_O_2_) and hydroxyl radicals (OH·) whereas proxF is selective for superoxide anions (O_2_^-^) and hydroxyl radicals, and their emission at 520 and 550 nm, respectively, increases upon exposure (Molecular Probe handbook). 6-weeks-old leaves were detached from plants grown at 120 μmol photons m^-2 ^s^-1^, 24°C, 8 h light/16 h dark, kept at 10°C, 25 μmol photons m^-2 ^s^-1 ^for 48 hours. Leaves were infiltrated with the dye solution (SOSG 5 μM, DCF 1 mM and proxF 1 mM) and illuminated with strong red light (λ>600 nm, 1600 μmol m^-2 ^s^-1^) at 10°C. We looked for increases in ROS-specific fluorescence to quantify ROS levels: SOSG (λ_exc _480 nm, λ_emis _530 nm); DCF (λ_exc _490 nm, λ_emis _525 nm); proxF (λ_exc _420 nm, λ_emis _515 nm).

### Extraction and measurements of metabolites

WT and *npq1lut2 *rosettes were pre-treated for 48 hrs at 10°C as above described, then were exposed to photoxidative conditions (1000 μmol photon m^-2 ^s^-1^, 10°C, 16 h light/8 h dark). Leaves were harvested and immediately frozen in liquid nitrogen at the same time of the day over a 6-day stress period. Plant material was ground to a fine powder in liquid nitrogen and either used immediately for assays or stored at -80°C. Ascorbate and glutathione were extracted and assayed following the method developed by Queval and Noctor [119]. ATP and ADP were assayed as previously described [120]. Amino acids and sugars were extracted and quantified as described by [121].

### In vivo fluorescence and NPQ measurements

Non-photochemical quenching of chlorophyll fluorescence (NPQ), maximum quantum efficiency of PSII (Fv/Fm) and photochemical quenching (qP) were measured with a PAM 101 fluorimeter (Walz, Effeltich, Germany) and were calculated according to [122]. Measurements were registered at the same hour every day over a 6-day-long stress treatment above described. For *in vivo *fluorescence measurements, leaves were illuminated for 25 min (1000 μmol photon m^-2 ^s^-1^, 10°C) and photosynthetic parameters were determined during steady-state photosynthesis.

### Pigment analysis

Pigments were extracted from whole leaves with 80% acetone (v/v), then separated and quantified by HPLC [10].

### Membrane isolation and thylakoid protein separation

Unstacked thylakoids were isolated from dark-adapted leaves or leaves treated with intense light as previously described [123]. SDS-PAGE analysis was performed with the Tris-Tricine buffer system [124]. Non-denaturing Deriphat-PAGE was performed following the method developed by Peter and Thornber [125,126]. For the identification of oxidized proteins, polypeptides were transferred to nitrocellulose membrane and carbonylated residues were identified by western blotting using the OxyBlot kit (Millipore). For immunoblot titration of CP47 (PsbC, PSII inner antennae), LHCII (Lhcb1, PSII outer antennae) and PsaA (PSI core complex), thylakoids corresponding to 0.5, 1, 2 and 4 μg of chlorophylls were separated by SDS-PAGE and the proteins detected by western blot with specific antibodies as described previously [20].

## Authors' contributions

AA carried out the molecular genetic studies and drafted the manuscript; LD carried out the biochemical and photosynthetic characterization of plants under photoxidative conditions, measurements of ROS and drafted the manuscript; PC carried out metabolomic analysis; AA. and ER participated in the RNA isolation, microarray experiments and statistical analysis of data, quantitative real-time PCR; LC and RB conceived the study and participated in its design and coordination. All authors read and approved the final manuscript.

## Supplementary Material

Additional file 1 **Tables describing WT and *npq1lut2 *transcriptome**. Click here for file

Additional file 2 **Figures describing WT and *npq1lut2 *plant photosynthetic characterization, transcriptome analysis and transcriptome validation**. Click here for file
