# Supplementary material for: Reactive oxygen species and transcript analysis upon excess light treatment in wild-type Arabidopsis thaliana vs a photosensitive mutant lacking zeaxanthin and lutein
Source: BMC Plant Biol. 2011 Apr 11;11:62. doi: 10.1186/1471-2229-11-62 (PMC3083342; doi:10.1186/1471-2229-11-62)
Supplement: Additional file 2 — Figures describing WT and npq1lut2 plant photosynthetic characterization, transcriptome analysis and transcriptome validation. [file 1471-2229-11-62-S2.DOC]

**Additional file 1**

**Figure S1. Gene clustering analysis.** A) Organization of all regulated genes in 11 clusters by using a k-means cluster analysis. There are small differences between the wild-type and *npq1lut2*. B) Clusters 1, 3, 13 and 18 highlighted the major differences between the two genotypes as detected by using a quality threshold algorithm (QT-clustering); in particular, cluster 18 showed the most evident differences between *npq1lut2* and wild-type plants and it is strongly enriched in chloroplast genes.

| **A**  **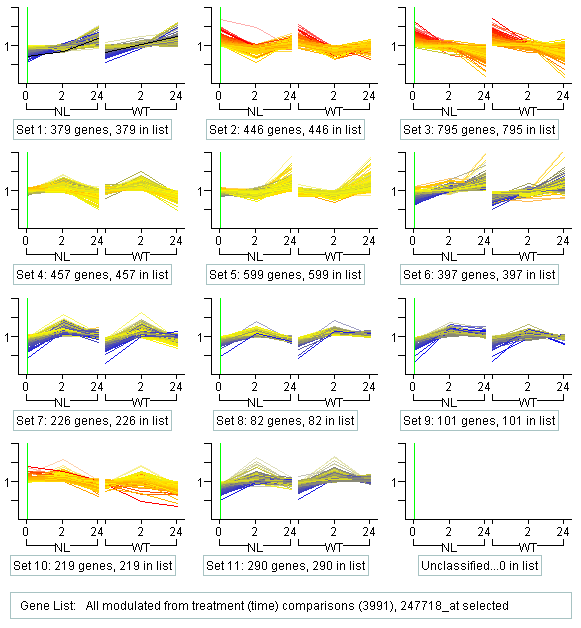** |
| --- |
| **B**  **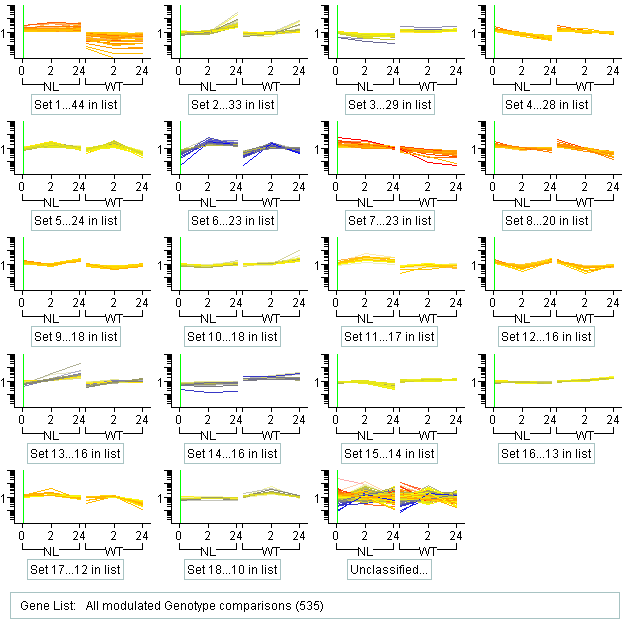** |

**Figure S2.** **Gene expression profiling by qRT-PCR and transcriptome validation.** The mRNA levels of four up-regulated genes (*Lhcb7*, *ELIP1*, *VTE1*, *DEFL*) and four down-regulated genes (*Lhca1*, *Lhcb4.2*, *CCD4*, *MYBL2*) were determined in WT and *npq1lut2* during the high light treatment by qRT-PCR and normalized with the expression level of the *UBQ10* reference. Measurements were performed in triplicates and for each gene the R^2^ is reported as the simple linear regression correlation coefficient between the qRT-PCR results and the values previously obtained through transcriptomics.

Primer sequences are the following: LHCb7 F 5’-TTATGCACAGGCTGCTTTCACT-3’, LHCb7 R 5’-GGATCCGAGACATGGTCAACA-3’; ELIP1 F 5’-GGCAGAGGCAAAGTCAAAAGG-3’, ELIP1 R 5’-GCAAACCGACCGTTCCATAG-3’; VTE1 F 5’-CAACACGCCCGAGCTACTAAA-3’, VTE1 R 5’-AAGGCGCTTTCAAGATCCAA-3’; DEFL F 5’-CGGAGGTGATGTTGGTTTCG-3’, DEFL R 5’-CTTGTGTAACAAATCGTCGGAAAT-3’; LHCa1 F 5’-GCGCTGTTGGCGTTTGTAG-3’, LHCa1 R 5’-GGTCCTGTCCCCGGGTAA-3’; LHCb4.2 F 5’-CGGTAAAGGACCGCTCAACA-3’, LHCb4.2 R 5’-GGTGGTGTGAAGTGGGTCACT-3’; CCD4 F 5’-GGAGAATCGAAGTTTCTGGTGATG-3’, CCD4 R 5’-CGGCGGCGACGATTT-3’; MYBL2 F 5’-CCTTCAGGACTCTAGCGAATCC-3’, MYBL2 R 5’-CATCGGAATAGAAGAAGCGTTTC-3’; UBI10 F 5’-GGTGGTTTCTAAATCTCGTCTCTGTT-3’ and UBI10 R 5’-GGGATTATACAAGGCCCCAAA-3’.


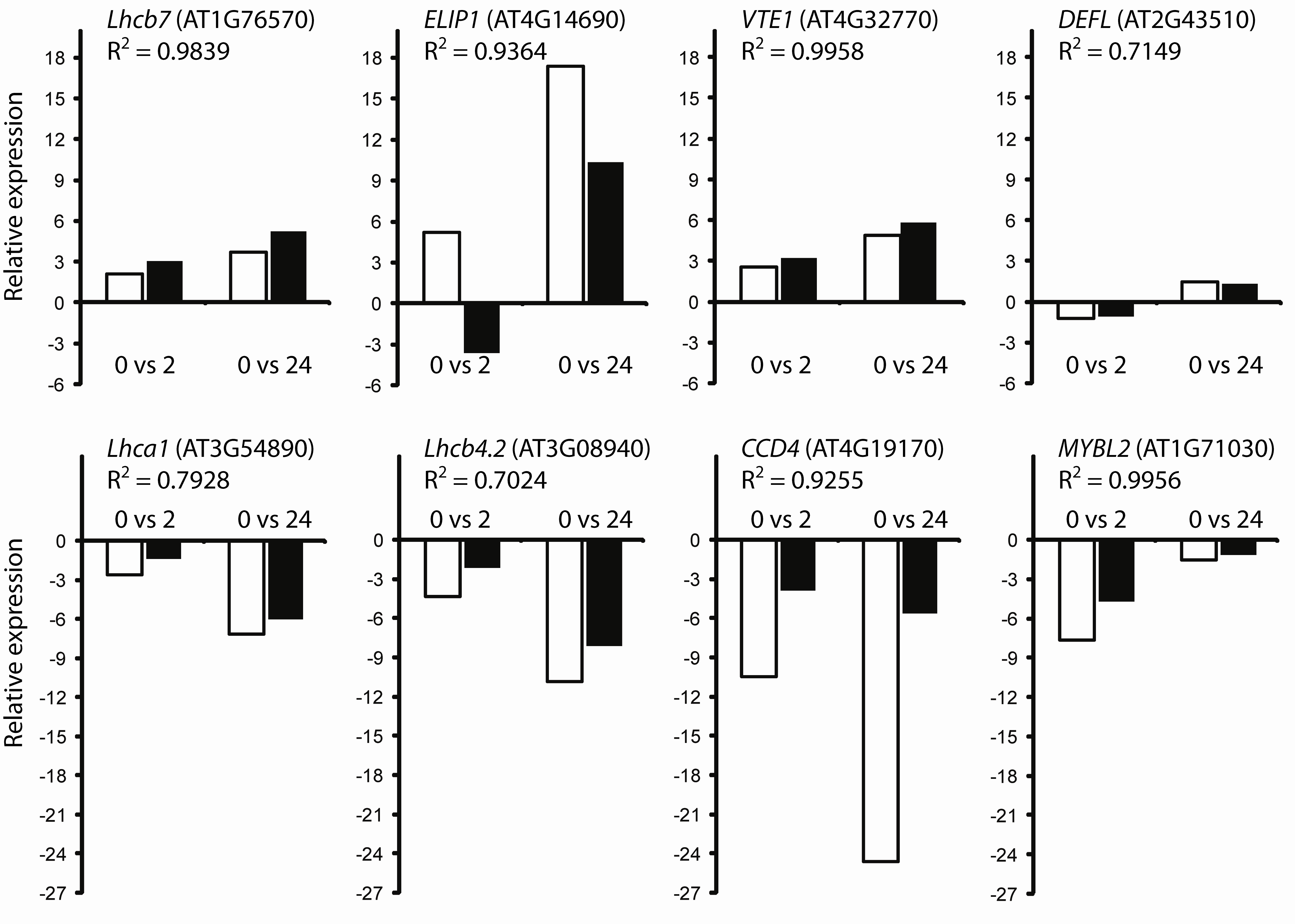


**Additional file 1: Figure S3. Time-course of chloroplast parameters putatively involved in the regulation of gene expression, as previously reported by Pfannschmidt et al. 1999, Walters 2005 and Piippo et al. 2006 [1-3].** Values that differ significantly between wild type and *npq1lut2* mutant plants (Student’s *t* test, p < 0.02) are marked by an asterisk.

**Figure S4. Comparison between the *npq1lut2* transcriptome (this work) and *flu*/*executer* transcriptome [4].** A) Statistical analysis of raw data. In Lee et al., plants grown under continuous light were kept for 8 hours in the dark and then put back to light for 30 minutes. In this experiment there is no time 0 (*i.e.* sample harvested before treatment) and therefore, while it is possible to identify genes differentially expressed between *flu*/*executer* and *npq1lut2* under stress conditions, it is not possible to define if transcription of a specific gene is activated or repressed. Here we are comparing the time points in the stress kinetic similar in the two experiments: 2 hours of treatment for *npq1lut2* and 30 minutes for *flu/executer*. The genes significantly up and down-regulated in the two genotypes after RMA analysis are reported. The conditions used in the two experiments are different, as judged from the high number of genes differentially expressed in the two wild-types (2420 genes). B) Venn diagrams showing the overlap between *npq1lut2* transcriptome and *flu/executer* transcriptome.


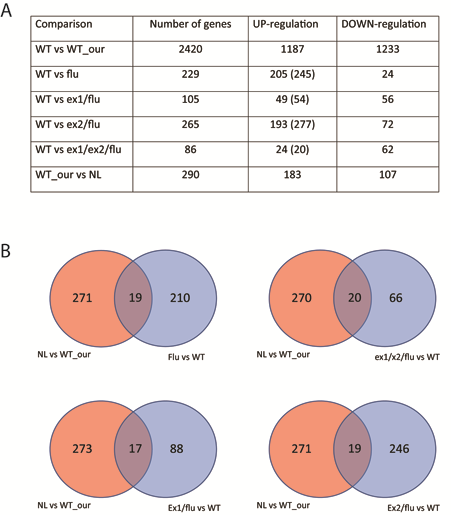


Reference List

1. Pfannschmidt T, Nilsson A, Allen JF: **Photosynthetic control of chloroplast gene expression.** *Nature* 1999, **397:** 625-628.

2. Piippo M, Allahverdiyeva Y, Paakkarinen V, Suoranta UM, Battchikova N, Aro EM: **Chloroplast-mediated regulation of nuclear genes in Arabidopsis thaliana in the absence of light stress.** *Physiological Genomics* 2006, **25:** 142-152.

3. Walters RG: **Towards an understanding of photosynthetic acclimation.** *Journal of Experimental Botany* 2005, **56:** 435-447.

4. Lee KP, Kim C, Landgraf F, Apel K: **EXECUTER1- and EXECUTER2-dependent transfer of stress-related signals from the plastid to the nucleus of Arabidopsis thaliana.** *Proceedings of the National Academy of Sciences of the United States of America* 2007, **104:** 10270-10275.
